# A prognostic stemness biomarker CCDC80 reveals acquired drug resistance and immune infiltration in colorectal cancer

**DOI:** 10.1002/ctm2.225

**Published:** 2020-10-31

**Authors:** Wei‐Da Wang, Guo‐Yan Wu, Kun‐Hao Bai, Ling‐Ling Shu, Pei‐Dong Chi, Si‐Yuan He, Xin Huang, Qian‐Yi Zhang, Liang Li, Da‐Wei Wang, Yu‐Jun Dai

**Affiliations:** ^1^ State Key Laboratory of Oncology in South China Guangzhou China; ^2^ Department of Hematologic Oncology Sun Yat‐sen University Cancer Center Guangzhou China; ^3^ Collaborative Innovation Center for Cancer Medicine Guangzhou China; ^4^ Department of Critical Care Medicine, Shanghai General Hospital Shanghai Jiao Tong University School of Medicine Shanghai China; ^5^ Department of Endoscopy Sun Yat‐sen University Cancer Center Guangzhou China; ^6^ The University of Texas MD Anderson Cancer Center UTHealth Graduate School of Biomedical Sciences Houston Texas USA; ^7^ National Research Center for Translational Medicine Ruijin Hospital Affiliated to Shanghai Jiao Tong University School of Medicine Shanghai China

To the Editor,

Colorectal cancer (CRC) is a malignance ranking the third cause of death in malignant tumors.^1^ The high mortality of CRC is mainly caused by frequent postoperative metastasis and multidrug resistance.^2^ A group of cancer stem cells (CSCs) have been discovered in CRC and the mRNA expression‐based stemness index (mRNAsi) was considered as a novel indicator to measure cancer development and drug resistance.^3^, ^4^ In the present study, we first explored the prognosis of mRNAsi in CRC patients and identified the critical genes related to immune infiltration and drug resistance.

The mRNAsi was much lower in normal samples than that in CRC tissues (*P* value = 7.458 × 10^−18^) and indicated a favorable prognosis in patients with CRC (*P* = .016), which was inconsistent with our general understanding in other type of cancers (Figure 1A and B).^5^, ^6^, ^7^ In addition, the mRNAsi had no relationship with clinical characteristics, including age, gender, tumor, node, metastasis (TNM) stage, and grade (Figure S1A). To explore the unique characteristic of mRNAsi in CRC, we performed the weighted gene co‐expression network analysis of 6501 differentially expressed genes and identified 17 modules (Figure 1C and Figure S1B and C). Among them, the brown, green, and yellow modules showed significant relationship with mRNAsi with correlation index −0.71 (*P* = 4 × 10^−55^), −0.71 (*P* = 8 × 10^−57^), and 0.71 (*P* = 1 × 10^−55^), respectively (Figure 1D). In total, 193 critical genes related to mRNAsi (135 genes in brown, 48 genes in green, and 10 genes in yellow) were filtrated (Figure 1E, Table S1). We found 24 key genes were significant closely associated with poor prognosis in CRC by univariate analysis (Figure 1F, Table S2). Causal relationship with proteins were analyzed by DisNor and indicated that these genes were closely connected and associated with adipogenesis (Figure 1G).

**FIGURE 1:**
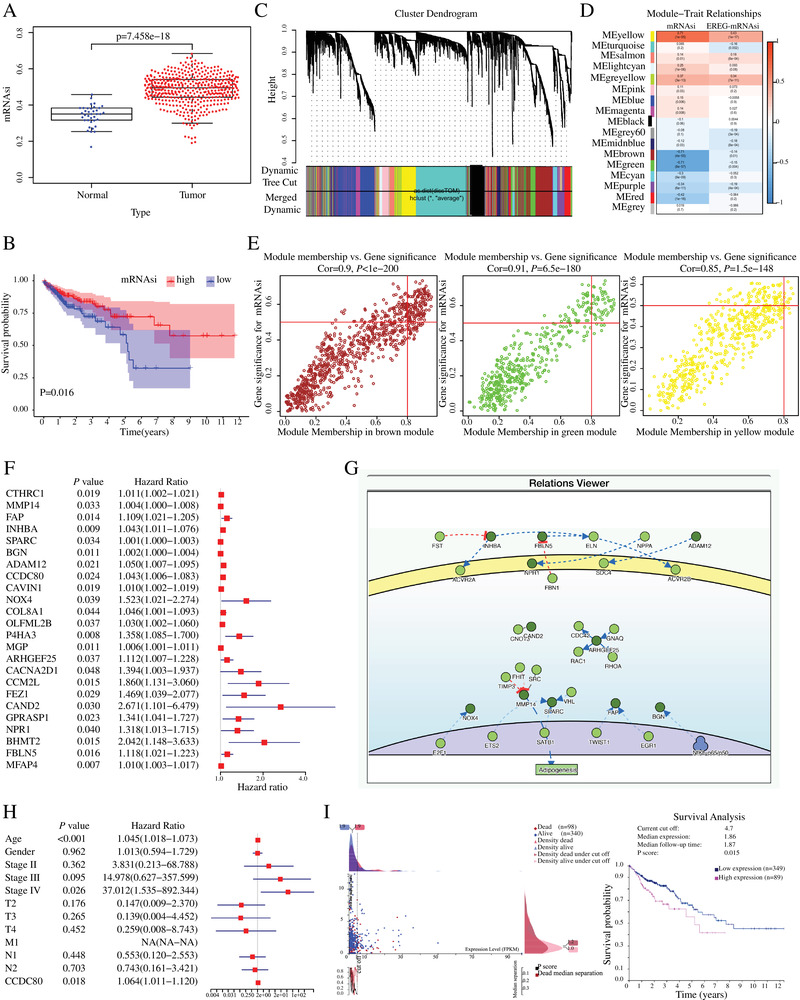
**Independent prognostic factors of mRNAsi and CCDC80 in CRC patients**. **A,** mRNAsi expression in normal (n = 32) and tumor (n = 375) samples. **B,** Overall survival of mRNAsi in CRC patients by Kaplan‐Meier analysis. **C,** WGCNA analysis of differentially expressed genes in CRC. Different colors correspond to related modules. D, Correlation coefficient between the modules and clinical traits with mRNAsi. *P*‐values are listed in the modules. **E,** Scatter plot analysis of different modules in the brown, green, and yellow modules. **F,** Univariate analysis of the key genes related to mRNAsi and the 24 significant genes were list. **G,** Signor analysis of 24 key genes in DisNor. **H,** Multivariate analysis of CCDC80 expression level with clinical characteristics in CRC patients. **I,** The cut‐off value and prognostic value of CCDC80 in CRC patients

Furthermore, multivariate analysis of expression levels of these 24 key genes with crucial clinicopathological parameters such as age, pathological stage, gender, and TNM stage (Table S3) showed that CCDC80 expression level was independent predictive factor (HR: 1.064, *P* = .018) (Figure 1H). Kaplan‐Meier analysis was performed based on the current cut off value calculated by Human Protein Atlas and indicated that higher the expression of CCDC80, the worsen is the prognosis for CRC patients (*P* = .015) (Figure 1I). The RNA and protein expression levels of CCDC80 in normal and tumor tissues were validated by the Pathology Atlas and UALCAN (Figure 2A and B).

**FIGURE 2:**
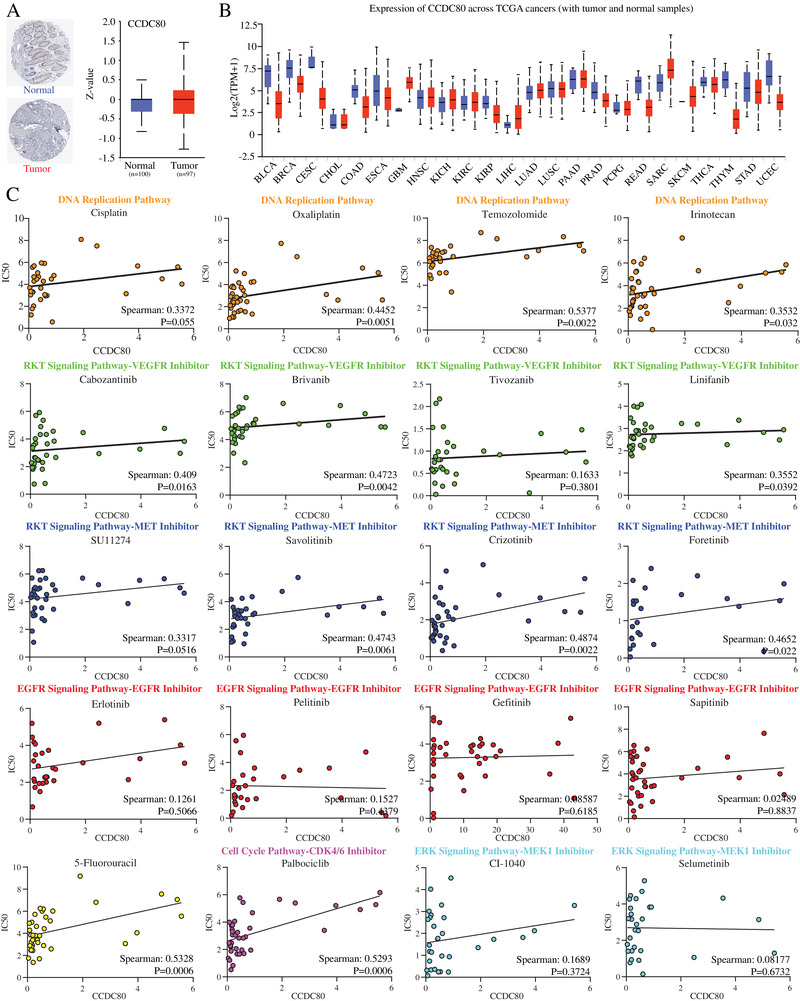
**A,** Immunohistochemistry of CCDC80 in normal and CRC tumor samples (left). The protein expression level of CCDC80 in normal and tumor samples from ULCAN database (right). **B,** Expression level of CCDC80 in normal tissues and pan‐cancer samples from TCGA database. **C,** The relationship of CCDC80 expression and chemotherapy resistance. Different colors indicate drugs involved in different pathways or targets

To explore the relationship between the drugs sensitivity and CCDC80 expression, the Genomics of Drug Sensitivity in Cancer database was utilized to analyze the correlation coefficient of CCDC80 level and IC50 of multiple drugs (Figure 2C). To our surprise, our data showed that the ectopic CCDC80 expression could induce resistance to the first‐line chemotherapy drugs including DNA replication pathway inhibitors and targeted therapeutic drugs such as VEGFR MET and CDK4/6 inhibitors, whereas the cancer cells with high expression of CCDC80 were still sensitive to EGFR and MEK inhibitors (Figure 2C).

Next, we overexpressed CCDC80 in Lovo cells by lentivirus infection and validated by RT‐PCR (Figure 3A). Consistent with the result in GDSC, the IC50 of CCDC80 overexpressed Lovo cells treated with 5‐fluorouracil, temozolomide, cabozantinib, crizotinib, or palbociclib, but not erlotinib, gefitinib, and selumetinib, was significantly increased compared with mock vector transduced group (Figure 3B). Furthermore, these drugs induced apoptosis of Lovo cells was partly declined by overexpressing CCDC80 (Figure 3C). Colony formation experiments suggested that overexpression of CCDC80 did not affect the number of colonies by treatment of erlotinib, gefitinib and selumetinib, but significantly increased the resistance to 5‐fluorouracil, temozolomide, cabozantinib, crizotinib, or palbociclib (Figure 3D). In addition, we further validated the effect of resistance to anticancer drugs by CCDC80 knockdown via a high CCDC80 expression cell line SW620, and reached a consistent conclusion (Figure 3B‐D).

**FIGURE 3:**
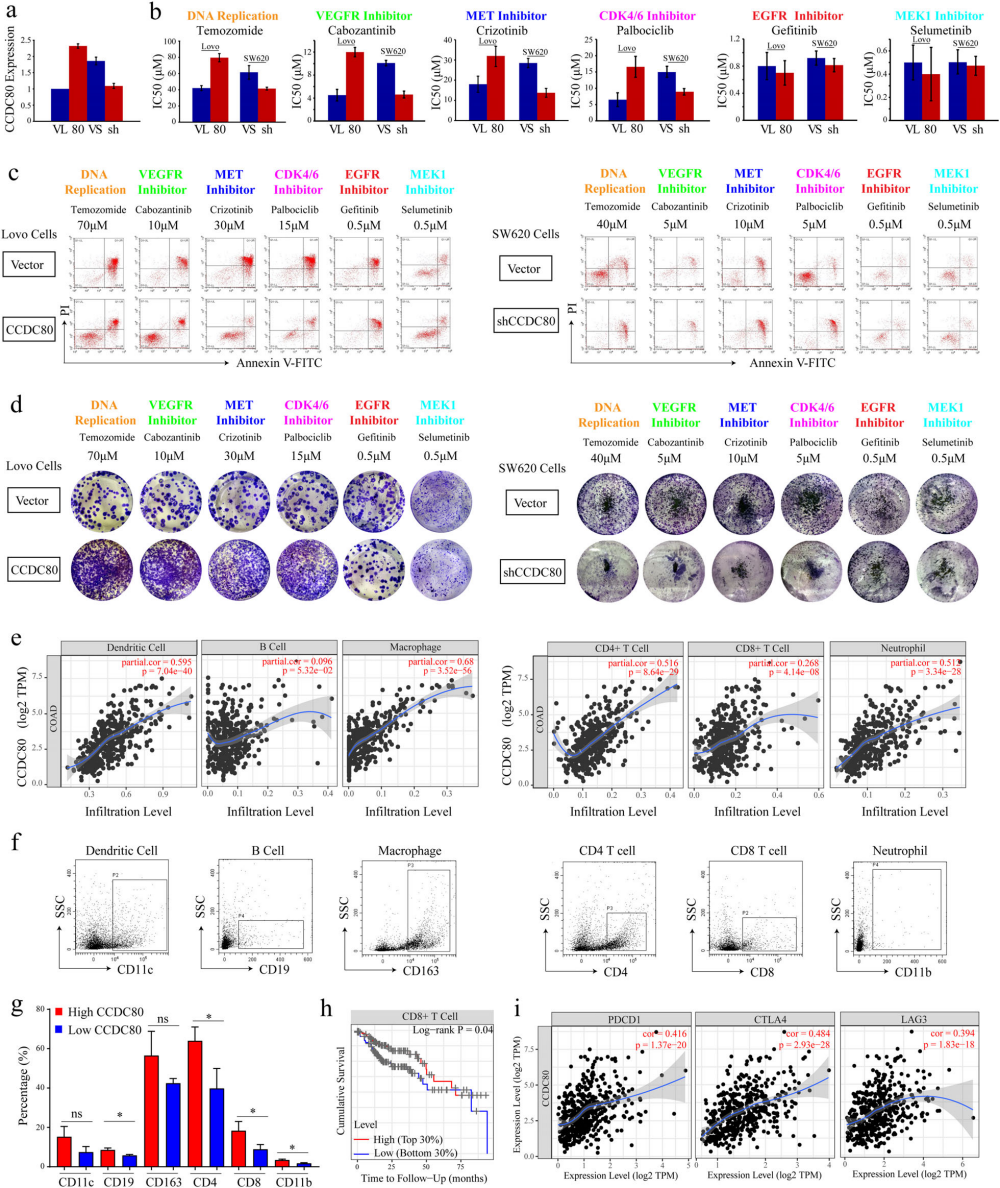
Acquired drug resistance and immune infiltration of CCDC80 in CRC. A, Results of RT‐PCR for CCDC80 in Lovo and SW620 cells transfected with vector, CCDC80 plasmid, or CCDC80 shRNA. B, IC50 of representative drugs in Lovo cells transfected with vector (VL) or CCDC80 (80) (left) and in SW620 cells transfected with vector (VS) or CCDC80 shRNA (sh) (right). C, Apoptosis induced by drugs in Lovo cells transfected with vector or CCDC80 (left) and in SW620 cells transfected with vector or CCDC80 shRNA (right). D, Clonal forming analysis of Lovo cells transfected with vector or CCDC80 (left) and SW620 cells transfected with vector or CCDC80 shRNA (right) treated with representative drugs. E, The correlation between the immune cells and the expression of CCDC80. F, Representative flow cytometry plots of infiltrated immune cells in patient samples. G, Statistical analysis of the percentage of infiltrated immune cells in primary samples. H, The correlation between CCDC80 expression and immunotherapy targets such as PD‐1, CTLA4, TIM‐3, and LAG‐3. I, The cumulative survival of CD8^+^ T cells in CRC samples

Tumor immunotherapy has made tremendous progress, changing the treatment pattern of many cancers.^8^, ^9^ We found a positive correlation of CCDC80 expression with the infiltration of B cells (*P* = 5.32 × 10^−02^), dendritic cells (*P* = 7.04 × 10^−40^), macrophage cells (*P* = 3.52 × 10^−56^), CD4^+^ T cells (*P* = 8.64 × 10^−29^), CD8^+^ T cells (*P* = 4.14 × 10^−08^), and neutrophil cells (*P* = 3.34 × 10^−28^) (Figure 3E). Furthermore, we validated the results by using patient samples and found that B cells, CD4^+^ T cells, CD8^+^ T cells, and neutrophil cells had a positive correlation with CCDC80 expression (Figure 3F and G). Among these cells, only CD8^+^T cells was significantly related to prognosis and the low level of CD8^+^ T cells indicated poor prognosis based on the top 30% and low 30% levels (Figure 3H and Figure S2). The targetable molecules for immunotherapy, such as PD‐1 (PDCD1), CTLA4, LAG3, and TIM‐3 (HAVCR2), were intensively associated with CCDC80 expression (Figure 3I, Table S4). Therefore, combination therapy of EGFR inhibitors or MEK inhibitors with immune checkpoint inhibitors may provide clinical benefits for CRC patients with high CCDC80 expression.

## CONFLICT OF INTEREST

The authors declare no conflict of interest.

## FUNDING

Guangci Distinguished Young Scholars Training Program; Grant Number: GCQN‐2019‐B17.

## AUTHOR CONTRIBUTIONS

Yu‐Jun Dai, Da‐Wei Wang, and Ling‐Ling Shu designed the concept and experiments. Wei‐Da Wang, Kun‐Hao Bai, and Guo‐Yan Wu performed the experiments; Wei‐Da Wang, Si‐Yuan He, Xin Huang, Qian‐Yi Zhang, Pei‐Dong Chi, and Liang Li collected the data and did the analysis. Wei‐Da Wang, Yu‐Jun Dai, and Guo‐Yan Wu prepared the manuscript draft. Yu‐Jun Dai and Da‐Wei Wang provided research support and revised the manuscript. All the authors approved the final proof.

## Supporting information

figureS1

figureS2

tableS1

tableS2

tableS3

tableS4

Supporting information
