# Supplementary figures and images for: A prognostic stemness biomarker CCDC80 reveals acquired drug resistance and immune infiltration in colorectal cancer
Source: Clin Transl Med. 2020 Oct 31;10(6):e225. doi: 10.1002/ctm2.225 (PMC7603297; doi:10.1002/ctm2.225)

A

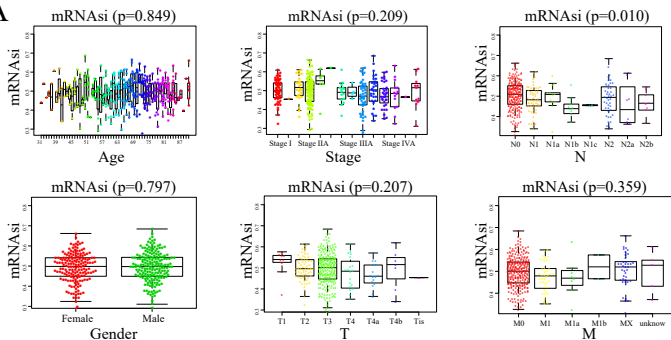

B

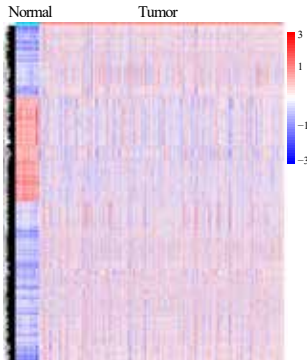

C

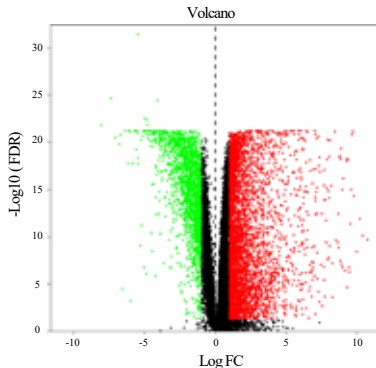

Supplement: Supplementary file 1 — figureS1 [file CTM2-10-e225-s001.pdf]

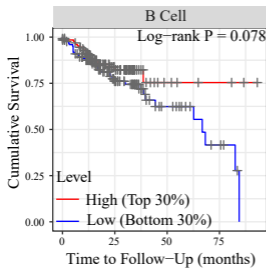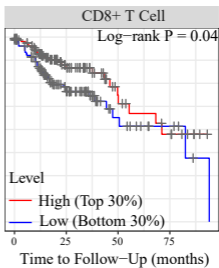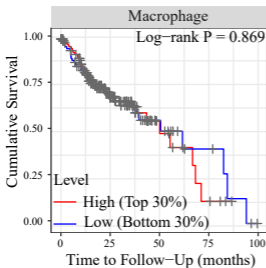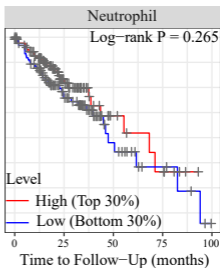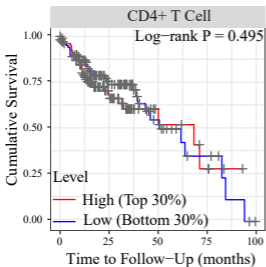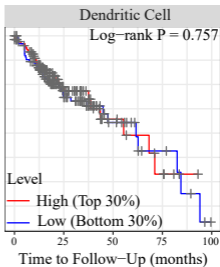

Supplement: Supplementary file 2 — figureS2 [file CTM2-10-e225-s002.pdf]
