# Supplementary material for: A prognostic stemness biomarker CCDC80 reveals acquired drug resistance and immune infiltration in colorectal cancer
Source: Clin Transl Med. 2020 Oct 31;10(6):e225. doi: 10.1002/ctm2.225 (PMC7603297; doi:10.1002/ctm2.225)
Supplement: Supplementary file 7 — Supporting information [file CTM2-10-e225-s007.docx]

**SUPPLEMENTAL INFORMATION**

**A Prognostic Stemness Biomarker CCDC80 Reveals Acquired Drug Resistance and Immune Infiltration in Colorectal Cancer**

Wei-Da Wang^1,2,6,†^, Guo-Yan Wu^3,†^, Kun-Hao Bai^1,4,6,†^, Ling-Ling Shu^1,2,6,†^, Pei-Dong Chi^1,2,6^, Si-Yuan He^5^, Xin Huang^1,6^, Qian-Yi Zhang^1,2,6^, Liang Li^1,2,6^, Da-Wei Wang^7,*^, Yu-Jun Dai^1,2,6,*^

1. State Key Laboratory of Oncology in South China, Guangzhou, 500026, China.

2. Department of Hematologic Oncology, Sun Yat-sen University Cancer Center, Guangzhou, 500026, China.

3. Department of Critical Care Medicine, Shanghai General Hospital, Shanghai Jiao Tong University School of Medicine, Shanghai, 200080, China.

4. Department of Endoscopy, Sun Yat-sen University Cancer Center, Guangzhou, 500026, China.

5. The University of Texas MD Anderson Cancer Center UTHealth Graduate School of Biomedical Sciences, Houston, TX 77030, USA.

6. Collaborative Innovation Center for Cancer Medicine, 651 Dongfeng East Road, Guangzhou, 500026, China.

7. National Research Center for Translational Medicine, Ruijin Hospital Affiliated to Shanghai Jiao Tong University School of Medicine, Shanghai 200025, China.

**Supplemental materials and methods**

**Patient Samples**

A total of 6 newly diagnosed CRC patients at the Ruijin Hospital Affiliated to Shanghai Jiao Tong University School of Medicine were enrolled in this study. All participants provided written informed consent in accordance with the regulations of the Institutional Review Boards of the Hospitals in agreement with the Declaration of Helsinki.

**Data Preparation**

In total, 32 normal samples and 375 colorectal cancer samples’ RNA-sequencing data were obtained from the TCGA database. We used a Perl script to merge the sequencing data of the CRC samples in a comprehensive matrix file. Ensembl IDs were converted to corresponding symbols by Ensembl database. Furthermore, we got the clinical data of 369 samples from the TCGA database. Next, we used the “edgeR” package to explore the differentially expressed genes (DEGs) between normal and CRC samples. A standard criterion was used to select the effective DEGs with fold change > 2; gene expression levels > 1 and false discovery rate (FDR) < 0.05.

**Weighted Gene Co-Expression Network Analysis**

We used the “WGCNA” R package to perform the functional analysis and the heterogeneity and accuracy of this network analysis were ensured by the strict selection of DEGs variance (25%). After filtering the outlier data of DEGs, we constructed the co-expression analysis by using Pearson correlation analysis and constructed the weighted adjacency matrix as previous reported with liver cancer analysis (1). A special b value was selected to raise the similarity matrix and to frame this co-expression network. Then, the adjacency matrix was converted to topological overlap matrix in the network to detect gene correlation. At last, we performed hierarchical clustering to construct the module dendrograms by utilizing the dissimilarity of topological overlap matrix.

**mRNAsi in Modules and Candidate Genes**

We performed a profiling molecular trait to get each sample’s molecular subtyping and its relationship with mRNAsi. The significant differences of the modules were analyzed by Kruskal–Wallis analysis. The colorectal cancer samples were divided into groups based on the mRNAsi index. The P value was used to evaluate the regression relationship between gene expression and the clinical data of colorectal cancer samples. We combined the modules with cutoff < 0.25 to heighten the consistency of the modules and analyzed the mRNAsi and epigenetically regulated mRNAsi with these gene modules. Finally, we set a threshold with module membership of correlation genes > 0.8 and gene significance of correlation genes > 0.5 to screen the candidate genes in modules. The overall survival analysis of mRNAsi index groups was evaluated by GraphPad Prism 8 software and the statistical analysis was performed by using Log-rank and unpaired t-test. The strength of the relationships among the key genes were analyzed by Pearson’s correlation analysis using the R corrplot package according to their gene expression levels.

**Signor Analysis**

We annotated the causal interaction data in Signor and explore the protein interaction net related to colorectal cancer with DisNor by using Mentha data (2).

**Prediction of Chemotherapeutic Response**

Genomics of Drug Sensitivity in Cancer (GDSC, [www.cancerrxgene.org](http://www.cancerrxgene.org)) was used to predict the chemotherapeutic response of colorectal cancer cell lines (3). This public pharmacogenomic database included a large number of drug sensitivity data of cell lines with all cancer types and cancer therapeutic related compounds. The IC50 of drugs were calculated by using “pRRophetic” package as described previously (4). The expression profiles of colorectal cancer cell lines were acquired in Cancer Cell Line Encyclopedia (CCLE, https://portals.broadinstitute.org/ccle/about) database (5). We merged the expression data of CCDC80 from CCLE and the drug sensitivity data from GDSC of all colorectal cancer cell lines into a comprehensive matrix file. After filtering the outlier data, we analyzed the correlation index between CCDC80 expression level and the IC50 for each chemotherapy drugs of colorectal cancer cell lines by using Spearman correlation analysis.

**Tumor Immune Infiltration Analysis**

Tumor Immune Estimation Resource (TIMER, <https://cistrome.shinyapps.io/timer/>) is a database contains the tumor infiltration data with immune cells including B cells, neutrophils, macrophages, CD8+ T cells, CD4+ T cells and dendritic cells (6). The correlation value between CCDC80 expression level and infiltration level of each type of immune cells was calculated automatically and determined by using the following guideline for the partial.cor value: 0.0-0.39 “weak”; 0.40-0.59 “mild”; 0.60-0.79 “strong”; 0.80-1.0 “very strong”. In addition, correlations between expression of CCDC80 and tumor-infiltrating immune-related genes were also explored by this module. The Kaplan-Meier curve parameters was split percentage of patients (30%) and survival time between 0 to 100 months. The prognosis value of immune cells in colorectal cancer was analyzed by Log-rank analysis with statistically significant (P < 0.05).

**The Human Atlas**

The expression level and survival value of CCDC80 was analyzed in the Human Atlas database. The cutoff values were defined according to the CCDC80 expression level (FPKM) and the time after diagnosis (years) and survival analysis was performed based on the cutoff values. The packages “ggplot2” and “survival” were applied to analysis the survival risk with univariate or multivariate. In addition, the Pathology Atlas provided the immune-histochemistry data of CCDC80 in normal tissue and colorectal cancer patients. The quantified expression levels of CCDC80 mRNA and protein in normal and cancer tissues were validated in UALCAN database (<http://ualcan.path.uab.edu/analysis.html>) (7).

**Plasmid Construction and Lentivirus-Mediated Infection**

The full-length human CCDC80 cDNA and CCDC80 shRNA were cloned into the lentivirus vector between NheI and NotI cloning sites. Lentiviruses expressing vector, CCDC80 cDNA or CCDC80 shRNA were co-transfected into 293T cells using Lipofectamine 3000 Transfection Reagent (Invitrogen) for lentivirus production. Lovo and SW620 cells were transduced with the lentiviruses for at least 48 h and sorted for GFP fluorescent positive cells. The target shRNA sequence was GGTGATTGTGTACGATTTAAT.

**Quantitative Real-Time RT-PCR**

The assays were performed according to instructions of the manufacturer (ESscience; QP002). The formula 2−ΔΔCt was used to analyze the data. CCDC80 Primers are Forward Primer: CCGCTCGGACATCAATGGG; Reverse Primer: GAAACGCAACATTCTTGACCG.

**Reagents**

All the Inhibitors Erlotinib (183321-74-6), 5-Fluorouracil (51-21-8), Cabozantinib (849217-68-1), Palbociclib (571190-30-2), Temozolomide (85622-93-1), Crizotinib (877399-52-5), Gefitinib (184475-35-2) and Selumetinib (606143-52-6) were obtained from TargetMol (Wellesley Hills, MA).

**Cell Viability and Apoptosis Assay**

The colorectal cancer cell lines (Lovo and SW620 cells) were obtained from Da-Wei Wang and was cultured in DMEM (Gibco, NY) with 10% fetal bovine serum (086-150, Multicell, South America). A density of 10^5^ cells per well was seeded into 96-well plates and incubated for 48 h. Then, 10 μL of CCK-8 (C0039, Beyotime, China) was added into each well and samples were measured at 450 nm absorbance by using a spectrophotometer. We used the apoptosis detection kit (FA111-02, Transgen Biotech) to detect apoptotic cells.

**Colony-Forming Cell Assays**

1000 cells were mixed with 2 mL medium and plated in a 6-hole plate culture dish. Colonies were photographed and dyed with crystal violet at day 14 after plating. All the experiments were performed for triplicate times.

**Flow Cytometric Analysis**

CRC primary cells for flow cytometric analysis were isolated from tissues grinded manually by using tissue strainer. Dissociated cells were suspended in buffer (PBS with 1% FBS) and stained with fluorochrome-conjugated antibodies: CD4-PE (Biolegend 317410), CD8-FITC (Biolegend 344704), CD19-APC (Biolegend 302212), CD11b-APC (Biolegend 301310), CD11c-FITC (Biolegend 301604) and CD163-PE (Biolegend 333606).

**Supplementary Figure Legends**

Figure S1. A. A comparison between mRNAsi expression level and clinical characteristics in CRC. B. Heatmap of differentially expressed genes between normal and CRC tissues. C. Volcano map of differentially expressed genes; red represent up-regulated genes, and green indicate down-regulated genes.

Figure S2. The cumulative survival of different infiltrated immune cells in CRC patients with high or low expression of CCDC80.

**Reference**

1. Bai KH, He SY, Shu LL, Wang WD, Lin SY, Zhang QY, et al. Identification of cancer stem cell characteristics in liver hepatocellular carcinoma by WGCNA analysis of transcriptome stemness index. Cancer Med. 2020.

2. Lo Surdo P, Calderone A, Iannuccelli M, Licata L, Peluso D, Castagnoli L, et al. DISNOR: a disease network open resource. Nucleic Acids Res. 2018;46(D1):D527-D34.

3. Yang W, Soares J, Greninger P, Edelman EJ, Lightfoot H, Forbes S, et al. Genomics of Drug Sensitivity in Cancer (GDSC): a resource for therapeutic biomarker discovery in cancer cells. Nucleic Acids Res. 2013;41(Database issue):D955-61.

4. Geeleher P, Cox N, Huang RS. pRRophetic: an R package for prediction of clinical chemotherapeutic response from tumor gene expression levels. PLoS One. 2014;9(9):e107468.

5. Bouhaddou M, DiStefano MS, Riesel EA, Carrasco E, Holzapfel HY, Jones DC, et al. Drug response consistency in CCLE and CGP. Nature. 2016;540(7631):E9-E10.

6. Li T, Fan J, Wang B, Traugh N, Chen Q, Liu JS, et al. TIMER: A Web Server for Comprehensive Analysis of Tumor-Infiltrating Immune Cells. Cancer Res. 2017;77(21):e108-e10.

7. Chen F, Chandrashekar DS, Varambally S, Creighton CJ. Pan-cancer molecular subtypes revealed by mass-spectrometry-based proteomic characterization of more than 500 human cancers. Nat Commun. 2019;10(1):5679.
